# The Application of Control Materials for Ongoing Quality Management of Next-Generation Sequencing in a Clinical Genetic Laboratory

**DOI:** 10.3390/medicina57060543

**Published:** 2021-05-28

**Authors:** Young-Kyu Min, Kyung-Sun Park

**Affiliations:** 1 Department of Medical Laser, Dankook University, Chungnam 31116, Korea; ykmin@yuhs.ac; 2 Department of Laboratory Medicine, Severance Hospital, 50-1 Yonsei-ro, Seodaemun-gu, Seoul 03722, Korea; 3 Department of Laboratory Medicine, Kyung Hee University School of Medicine and Kyung Hee University Medical Center, Seoul 02447, Korea

**Keywords:** next-generation sequencing, quality control, control material, multiplexing cell lines

## Abstract

Next-generation sequencing (NGS) has played an important role in detecting genetic variants with pathologic and therapeutic potential. The advantages of NGS, such as high-throughput sequencing capacity and massively parallel sequencing, have a significant impact on realization of genetic profiling in clinical genetic laboratories. These changes have enabled clinicians to execute precision medicine in diagnosis, prognosis, and treatment for patients. However, to adapt targeted gene panels in diagnostic use, analytical validation and ongoing quality control should be implemented and applied with both practical guidelines and appropriate control materials. Several guidelines for NGS quality control recommend usage of control materials such as HapMap cell lines, synthetic DNA fragments, and genetically characterized cell lines; however, specifications or applications of such usage are insufficient to guideline method development. This review focuses on what factors should be considered before control material selection for NGS assay and practical methods of how they could be developed in clinical genetic laboratories. This review also provides the detailed sources of critical information related to control materials.

## 1. Introduction

Next-generation sequencing (NGS) has had a drastic impact on clinical genetic laboratories involved in detecting mutations and clonal heterogeneity, therapeutic decision making, monitoring therapy response, and disease prediction for at-risk patients through technical strength [1,2,3,4].

With the advantages of NGS technology such as massively parallel sequencing, high-throughput sequencing data, and low sequencing cost per base, clinicians are able to provide more rapid diagnoses and patients would receive adequate treatment. However, this only becomes possible under the assurance of complete quality control (QC) similar to other molecular methods. QC of NGS method is more challenging because NGS data is generated through complex protocols [5]. NGS workflow is a two-step process: wet- and dry-bench. The wet-bench process is composed of nucleic acid isolation, library preparation, and sequencing; whereas the dry-bench process is a bioinformatic data analysis starting from mapping sequence reads to a reference genome to searching for variants that are clinically meaningful.

Because of these complexities, the College of American Pathologists (CAP) checklist requires specific quality management programs that specify controls, metrics, and quality control parameters to monitor overall procedures in a clinical laboratory [6]. To ensure accuracy of the patient’s genetic test results, these quality management programs should be adhered to in every clinical run [2,6,7]. Control materials for QC should be used in assay validation or revalidation, assessment of analytical performance, reagent lot change, the comparison of inter-laboratory (for external proficiency test) or between-operators (for internal proficiency test). In selecting control materials, several points should be considered: control materials should be interchangeable to clinical specimens; control materials for the targeted panel should represent genetic variants that are detectable, and detected variants should be concordant to known allele fractions. However, it is practically difficult to obtain well-characterized control materials that perfectly fit in all considerations. To overcome this difficulty, the CAP and Association for Molecular Pathology (AMP) recommend error-based approach to determine potential sources of error that could occur through all steps of NGS method [8]. These errors could be revealed during assay design, assay validation, and/or QC, such as the quality of nucleic acids from the several sources of a patient’s specimen and variant calling error in a technically limited region, etc. Focusing on the control materials for QC in clinics, this review will summarize considerations when choosing control materials for targeted NGS panels and suggest adequate control materials according to the type of targeted panels and methods for design.

## 2. Considerations of Control Material Selection

### 2.1. Sample Characteristics

**Figure 1:**
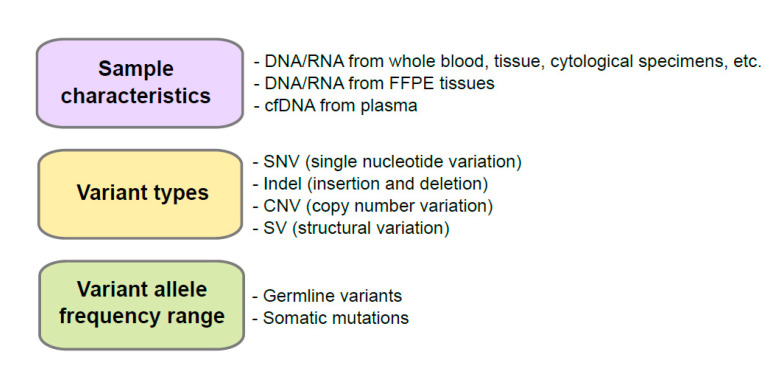
Consideration before development of control materials. FFPE, formalin-fixed and paraffin-embedded; cfDNA, cell-free DNA.

We summarized the factors to consider when selecting control materials in Figure 1. The controls to ensure adequate nucleic acid extraction during the analytical wet bench process are needed. This control is necessary because quality and quantity vary from the source of a patient’s specimen and the accuracy of NGS data is influenced by the purity, quantity, and quality of nucleic acid used [7]. NGS platforms have different clonal amplification and sequencing chemistries such as sequencing by synthesis (e.g., Illumina NGS platforms, Illumina, San Diego, CA, USA) and Ion semiconductor sequencing (e.g., Ion systems, ThermoFisher, Waltham, MA, USA) [8]. Especially, the amplicon-based enrichment methods (e.g., Ion Torrent series, ThermoFisher, Waltham, MA, USA) requires less input DNA/RNA than hybrid capture-based enrichment methods (e.g., Illumina series, Illumina, San Diego, CA, USA) [8]; therefore, DNA/RNA input requirements is the one of important factors to determine which platforms to use. Although the amplicon-based enrichment methods require less input DNA/RNA, they are vulnerable to chemistry issues such as allele dropout and primer mismatches [8].

Nucleic acids extracted from whole blood, tissue, or cytological specimens such as direct smear, liquid based cytology, or supernatant are usually used for genetic tests. If these samples are not in a low-cellularity status, high-quality DNA or RNA can be extracted [9]. However, solid tissues, bone marrow, or cytologic specimens for cell blocks are in many cases formalin-fixed and paraffin-embedded (FFPE), a standard procedure to preserve samples in room temperature, damaging nucleic acids including formaldehyde-induced crosslinks, fragmentation of DNA (fragment size ranging from ~180 bp to ~3000 bp), and deamination of cytosine bases causing C to T mutations that result in the low quality and yield of nucleic acids [10]. Cell-free DNA (cfDNA) is a liquid biopsy specimen that circulates in the bloodstream caused by apoptosis or necrosis, but maintains the remarkable average fragment length of around ~180 bp [11,12]. Noninvasive prenatal testing, such as screening chromosomal aneuploidies, especially for trisomy and/or monosomy by analyzing fetal cfDNA from a maternal blood specimen, is an example used in clinics that utilizes cfDNA [13,14]. Profiling of genetic-alteration in cfDNA is also applied in cancer diagnosis in early stages, monitoring response to therapy, and the prediction of minimum residual disease [12]. Because test results may vary depending on the type of patient’s specimen used, control materials should be the same or similar to the conditions of patient’s specimen and mimicking process is necessary.

### 2.2. Variant Types

To develop control materials for a targeted gene panel for a specific disease, prevalent pathogenic variants and their types for the tested genes need to be well characterized and considered. To confirm which variant types compose the pathogenic variants in a specific disease, it is necessary to review databases for genetic variants or related papers. Some examples include ClinVar (https://www.ncbi.nlm.nih.gov/clinvar, accessed on 21 April 2021), Online Medelian Inheritance in Man (OMIM, https://www.omim.org, accessed on 21 April 2021), GeneReviews (https://www.ncbi.nlm.nih.gov/books/NBK1116, accessed on 21 April 2021), the Human Gene Mutation Database (http://www.hgmd.cf.ac.uk/ac/index.php, accessed on 21 April 2021), Leiden Open Variation Database (https://databases.lovd.nl/shared/genes, accessed on 21 April 2021), Database of genomic variation and Phenotype in Humans using Ensembl Resources (DECIPHER, https://www.deciphergenomics.org, accessed on 21 April 2021), or other locus specific databases, etc. for germline variants and Catalogue Of Somatic Mutations In Cancer (COSMIC, https://cancer.sanger.ac.uk/cosmic, accessed on 21 April 2021), The Cancer Genome Atlas (TCGA, https://portal.gdc.cancer.gov, accessed on 21 April 2021) and International Cancer Genome Consortium (ICGC) Data Portal (https://dcc.icgc.org, accessed on 21 April 2021), etc. for somatic mutations.

For QC of variant calling step in the bioinformatics process, not only variant types across the spectrum (single nucleotide variant (SNV), insertion and deletions (indels), copy number variant (CNV), and structural variant (SV)) but also the characteristics of variants’ regions (repeat region, homopolymer region, and GC rich region, etc.) need to be considered [2]. Although SNVs and small indels can be detected well using NGS assay, detecting large indels, variants located in the homopolymer or repeat region, detection of CNVs or SVs remain as challenge [15,16]. Moreover, the types of NGS platforms are also considered in the bioinformatic process [8]. Illumina platforms require more exquisite bioinformatic analysis; however, error rate in homopolymer regions is increased in Ion Torrent series [8].

### 2.3. Variant Allele Frequency Range

Due to the diversity of genetic variation including germline variants and somatic mutations in humans, the targeted NGS panel is designed according to the specific target-genes and their genetic characteristics. Panel for germline variants is implemented to detect associated genes with particularly hereditary variants in families [17,18]. Diploid zygosity can be calculated with the percentage of sequencing read called variant allele frequencies (VAFs) using NGS technology [7,19]. Germline variants have three expected values (excluding mosaicism) of VAFs: VAF of homozygous reference allele is near 0%, heterozygous allele is near 50%, and homozygous alternate allele is near 100%. For somatic mutations, VAF is unpredictable because population of tumor cells compared to normal cells can vary sample to samples. Qualitative as well as quantitative QC is crucial for somatic mutations especially for precision of assay.

## 3. Materials and Applications for Quality Control

### 3.1. Controls for Germline Variants

We summarized control materials for next-generation sequencing in Table 1. Clinical genetic laboratories use certified or standard reference materials such as haplotype map (HapMap) samples for panel validation and ongoing QC for the detection of germline variants. Genome in a Bottle (GIAB) consortium (https://www.nist.gov/programs-projects/genome-bottle, accessed on 21 April 2021) from National institute of Standards and Technology (NIST) produced high-confidence variant sets for HapMap samples such NA12878, NA24385, NA24631, and made them publicly available. [20,21]. With these public data, the gold standard reference variant call from the GIAB (https://www.nist.gov/programs-projects/genome-bottle, accessed on 21 April 2021) could be compared with experimental NGS data [22]. Recently, GIAB has also released germline SV benchmark [23] and small variant benchmark for more difficult regions to call variants in (https://www.biorxiv.org/content/10.1101/2020.07.24.212712v3, accessed on 21 April 2021). Cell lines or DNA for NIST reference samples can be obtained from the Coriell Institute (https://www.coriell.org/1/NIGMS/Collections/NIST-Reference-Materials, accessed on 21 April 2021).

Validation process for detecting germline variants in certain hereditary diseases or conditions should include samples with representing pathogenic variants. Genetic Testing Reference Materials Coordination Program from Centers for Disease Control and Prevention (https://www.cdc.gov/labquality/get-rm/index.html, accessed on 21 April 2021), Coriell Institute Human Genetic Cell Repository (https://www.coriell.org/1/NIGMS, accessed on 21 April 2021), European Collection of Authenticated Cell Cultures (https://www.phe-culturecollections.org.uk/collections/ecacc.aspx, accessed on 21 April 2021) or commercial reference materials (CRMs) such as SeraCare (https://www.seracare.com/Controls---Reference-Materials-NGS-Inherited-Disease, accessed on 21 April 2021) or Horizon Diagnostics (https://horizondiscovery.com/en/reference-standards, accessed on 21 April 2021) are helpful in getting the information of reference materials for specific hereditary genetic disorders as well as human leukocyte antigen testing or pharmacogenetics. Although there are various information sources and reference materials for hereditary genetic diseases, it is still challenging to get adequate reference materials for rare diseases, as multiplexing cell line-based reference materials to include the variety of clinically relevant pathogenic variants continuously dilutes VAF. Genetic variants detected in patients’ samples that are validated by orthogonal molecular methods such as Sanger sequencing, multiplex ligation-dependent probe amplification, or quantitative polymerase chain reaction (PCR) can also be used as means of quality control. However real patients’ samples are finite in their amount compared to cell lines; therefore, both cell lines and real specimens are used together to validate the clinical performance of customized targeted panels and only cell lines are sequenced for ongoing QC in routine clinical runs [24].

### 3.2. Controls for Somatic Mutations

In a clinical laboratory, cancer cell lines or CRMs are usually used for validation and/or QC for detecting somatic variants due to ease of access and applicability. Somatic mutations in cancer cell lines are well-defined in COSMIC (https://cancer.sanger.ac.uk/cell_lines, accessed on 21 April 2021) or Cancer Cell Line Encyclopedia (CCLE) (https://portals.broadinstitute.org/ccle, accessed on 21 April 2021). Cancer cell lines can be obtained through American Type Culture Collection (https://www.atcc.org/Products/Cells_and_Microorganisms/Cell_Lines/Human.aspx, accessed on 21 April 2021), or DSMZ (https://www.dsmz.de/collection/catalogue/human-and-animal-cell-lines/catalogue, accessed on 21 April 2021). However, true VAF of heterozygous variants in cancer cell lines is unclear because it may be influenced by genomic instability and drift.

The CRMs for NGS that are accessible at Horizon Diagnostics (https://horizondiscovery.com/en/reference-standards, accessed on 21 April 2021), and SeraCare (https://www.seracare.com/Controls---Reference-Materials-NGS, accessed on 21 April 2021) are specifically designed using gene editing and gene modulation. The CRMs include somatic hotspot mutations which allele frequencies are validated by absolute quantification method. However, CRMs are not cost-effective to use for continuous QC in routine clinical runs.

In-silico data sets can be used to validate bioinformatic analysis: some example studies include benchmark somatic variant calls and regions from the study by Lee AY et al. [25] (https://github.com/adamewing/bamsurgeon, accessed on 21 April 2021) or the study by Craig DW et al. [26] (database of Genotypes and Phenotypes; accession number phs000932).

### 3.3. Multiplexing HapMap and Well-Characterized Cancer Cell Lines for Somatic Mutations

**Figure 2:**
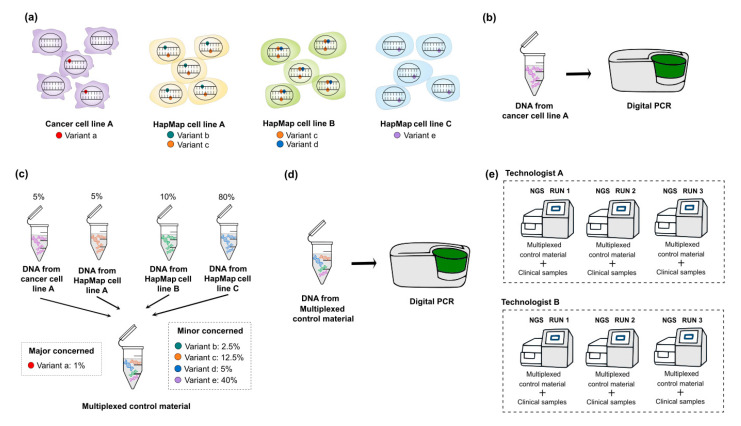
Schematic diagrams of developing multiplexed control materials to detect somatic variants. To multiplex HapMap and well-characterized cancer cell lines, following steps are necessary. (**a**) Selection of target genes and variants which are expected to be detected from both HapMap and cancer cell lines. In this step, major concerned variants (hotspot mutations) and minor concerned variants are defined by referring to the public database (e.g., GIAB for HapMap cell lines and COSMIC or CCLE for cancer cell lines). (**b**) Quantification for the allele frequency of major concerned variants in cancer cell lines by digital PCR. Before multiplexing cell lines, the quantification of the selected cancer cell lines is essential because the exact allele frequency of heterozygous variants in the cancer cell lines is unknown. (**c**) Multiplexing HapMap and cancer cell lines. To include the maximum number of minor concerned variants with various allele frequency ranges, the calculation for mixing ratio is necessary. (**d**) Quantification for the allele frequency of expected major concerned variants in developed multiplexed reference materials. (**e**) Establishing an answer set of detected variants with their VAF ranges and the list of variants that are presumed to be background errors through the repetition of NGS assay. Multiplexed reference materials should be sequenced with clinical samples. GIAB, Genome in a Bottle; COSMIC, Catalogue of Somatic Mutations in Cancer; CCLE, Cancer Cell Line Encyclopedia; VAF, variant allele frequency; NGS, next-generation sequencing.

Although CRMs are easily accessible, the representative mutations of customized targeted panel are highly limited. Because somatic mutations in tumors include hotspot mutations such as *BRAF* V600E mutation or *JAK2* V617F and non-hotspot mutations covering the whole coding or non-coding regions such as *TP53* mutation, the identification of systemic error is important for the accuracy of test. Therefore, multiplexing HapMap cell lines can be one of the options to develop a control material including variants with expected allele frequencies [8]. However, genetic variants from HapMap cell lines [27,28] are validated as germline variants. Alternatively, multiplexing both HapMap cell lines (for minor concerned variants) and well-characterized cancer cell lines (for major concerned variants) are recommended to develop control materials (Figure 2). Major concerned variants are hotspot mutations which are used to validate accuracy, precision and the limit of detection (LOD); whereas, minor concerned variants are non-hotspot mutations which are used to detect systemic error and validate precision indirectly. Especially, hotspot mutations located within target genes that have the lowest coverage by NGS can be selected and VAFs of these mutations can be set as low as the LOD. This is an error-based approach; if the alternative read depth of targeted variant is certainly enough to be called through pipeline, the analytical sensitivity of genetic alteration is assured [29,30,31].

The quantification of selected cancer cell line before dilution is essential because the allele frequency of heterozygous variant in the cell line is unknown; absolute quantification can be accomplished with digital PCR, a new generation PCR technique with high precision and sensitivity to detect target sequences [32,33,34]. In terms of quantitative aspects, all selected mutations should be confirmed by absolute quantification method for the accuracy of NGS data. However, it is not cost-effective to validate all mutations in a clinical genetic laboratory, so we recommend to validate the exact allele frequency of selected major concerned variants by digital PCR, and to establish answer set for the allele frequency range of minor concerned variants through the repeated sequencing of NGS assay.

To multiplex HapMap and a well-characterized cancer cell line, the following steps are necessary: first, the selection of target genes and hotspot mutations (consider including multiple types of variants), next selecting well-characterized cancer cell lines that contain expected mutations in databases (COSMIC, CCLE, GIAB), after that, quantifying the allele frequency of major concerned variants included in selected cancer cell lines by digital PCR, then, calculating the correct ratio of the cell lines to mix, culturing and extracting nucleic acids from the selected cancer cell line, diluting of cancer cell line to HapMap cell line to make desired allele frequency (set the VAF of hotspot mutations as low as the LOD level), then mimicking FFPE or cfDNA if necessary, quantifying the allele frequency of expected major concerned variants in developed control material by digital PCR, and finally, establishing an answer set (VAF range for detected non-hotspot and hotspot mutations) through NGS assay. We simplified the schematic diagrams of developing multiplexed reference materials to detect somatic mutations in Figure 2.

In order to include the maximum number of minor concerned variants with various allele frequency ranges, it is recommended to consider the following: increasing the ratio of cell lines that have fewer mutations, leads of reduction of ratio of cell lines that have more homozygous mutations [35]. It is also necessary to ensure that other CNVs do not exist in the region where the selected hotspot mutation by bioinformatic analyses before multiplexing. If CNVs exist, there would be a difference between expected and experimental allele frequency. Especially, the precision of quantitative detection is essential to monitor the true increase/decrease of mutational clone distinguishing analytic fluctuations. The precision can be carried out by targeting both major and minor concerned variants in control materials. Since the percentages of the coefficient of variation are increased as the allele frequency of mutations is decreased, it is necessary to adjust target sequencing depth to achieve acceptable precision [35].

### 3.4. Mimicking Process

For mimicking FFPE specimen, cell line pellet needs to be fixed and embedded by formalin and paraffin as patient’s tissue specimen is processed [36]. A previous study used only 4% paraformaldehyde and fixation at 4 °C for 20 min to model a similar stimulus to the control material [37]. For mimicking cfDNA specimen, it is important to produce the average of around ~180 bp length fragment of DNA. This could be accomplished by either PCR assay or physical fragmentation. For a PCR-based method, targeted regions should be verified by orthogonal molecular methods such as Sanger sequencing to confirm the amplification of expected mutation and the target PCR product should be purified by gel-electrophoresis [37]. Fragmented DNA can also be made by a shearing method using an ultra-sonication technique. The resultant shorter DNA fragment is spiked to pooled plasma, which should be confirmed by digital PCR to not include targeted mutation. After genomic DNA is extracted using the same kit and method as that of patient sample, target mutation should be quantified by digital PCR to compare expected and experimental allele frequency once again. If the allele frequency of targeted mutations is acceptable, control materials should be tested with NGS to establish an acceptable range before application to quality monitoring routine.

### 3.5. Synthetic DNA or Engineered Cell Line by Gene-Editing

Synthetic DNA (plasmid-based materials) is another option to supplement the use of multiplexing cell lines for control material [38,39,40]. Although the synthetic DNA is not equivalent to patient’s specimen because of its inability to represent the full size of genome and lack of nucleic acid extraction step, this approach has several advantages over the previous methods. Desired target mutations can be easily engineered into human genomic DNA, and the amount of target mutations and their VAF can be easily controlled (covering germline variants and somatic mutations). Molecular barcodes can also be used to verify that mutation sequence originated from synthetic DNA not from normal control DNA [38,40].

The reference materials using engineered cell line by gene-editing (e.g., by CRISPR/Cas9) is also another option [41]. They include the context of full size of genome and are highly interchangeable with patient samples. However, it is not easy to use in a clinical genetic laboratory setting due to many technical difficulties such as off-target effects.

## 4. Conclusions

In this review, we have discussed the considerations to select adequate control materials for NGS assay, the type of QC materials, and how they could be developed in clinical laboratories. Although several previous review studies have discussed the types of control materials for NGS and their advantages and disadvantages, however, in this review, we focused on practical aspects of selecting and developing NGS QC materials in clinical genetic laboratories, explaining what we consider and need to understand, so that they can be applied directly it in their clinical laboratories. In addition, because the sources of critical information for the development of NGS QC are scattered, it is difficult to obtain such information in genetic laboratories that do not have sufficient experience, so we intended to describe it as much detail as possible. Although assay design and validation are performed according to the standard guidelines in clinics, screening of systemic errors with adequate control materials that potentially occur in routine runs is highlighted. To verify the entire process of NGS assay for accuracy, precision, analytical sensitivity, analytical specificity, and the LOD, this review has introduced several options for control materials. Each control material has pros and cons. Clinical laboratories should develop quality management programs according to the purpose and characteristics of NGS assay.

## Figures and Tables

**Table 1:** Overview of control materials for next-generation sequencing.

Purpose	Control Materials	Characteristics	Sources
Detecting germline variants	Standard reference materials (e.g., HapMap reference materials)	Highly interchangeable with patient samples A set of high-confident variant calls is published Only validated as germline variants Specific genetic diseases or conditions are not reflected	Coriell Institute (https://www.coriell.org/1/NIGMS/Collections/NIST-Reference-Materials, accessed on 21 April 2021) Genome in a Bottle (https://www.nist.gov/programs-projects/genome-bottle, accessed on 21 April 2021)
	Reference materials for hereditary genetic disorders or conditions	Highly interchangeable with patient samples Specific genetic diseases or conditions are reflected One reference material contains a limited number (one or two) of (likely) pathogenic variants Variants are limited	Genetic Testing Reference Materials Coordination Program from Centers for Disease Control and Prevention (https://www.cdc.gov/labquality/get-rm/index.html, accessed on 21 April 2021) Coriell Institute (https://www.coriell.org/1/NIGMS/Collections/Heritable-Diseases, accessed on 21 April 2021) European Collection of Authenticated Cell Cultures (https://www.phe-culturecollections.org.uk/collections/ecacc.aspx, accessed on 21 April 2021)
Detecting somatic mutations	Cancer cell lines	Highly interchangeable with patient samples Cancer cell line is inexhaustible VAF of heterozygous variants is unclear and influenced by genomic instability and drift Absolute quantification step is essential for quantitative QC	American Type Culture Collection (https://www.atcc.org/Products/Cells_and_Microorganisms/Cell_Lines/Human.aspx, accessed on 21 April 2021) DSMZ (https://www.dsmz.de, accessed on 21 April 2021) Catalogue Of Somatic Mutations In Cancer (https://cancer.sanger.ac.uk/cell_lines, accessed on 21 April 2021) Cancer Cell Line Encyclopedia (https://portals.broadinstitute.org/ccle, accessed on 21 April 2021)
	Multiplexing reference materials (multiplexing HapMap and well-characterized cancer cell lines)	Error-based and cost-effective approach considering qualitative and quantitative QC for routine clinical runs Representative mutations can be validated with major concern variants Wide range of VAF can be monitored with minor concern variants Absolute quantification step is essential for major concern variants	
Detecting germline variants or Somatic mutations	Synthetic DNA or engineered cell line by gene-editing	Disease-specific variants with expected allele frequency can be reflected as intended Wide range of variant types are covered (e.g. SNV, indel, CNV, gene fusion, etc.) Not commutable with patient samples when using synthetic DNA method In order to develop high-quality QC materials, the sufficient experience of experiment in the clinical laboratory is needed	
	Commercial reference materials	Clinically relevant variants are validated Wide range of variant types are covered (e.g. SNV, indel, CNV, gene fusion, etc.) Ready to use Not cost-effective to be applied in QC in routine clinical runs Variants and their allele frequencies are limited	Horizon Diagnostics (https://horizondiscovery.com/en/reference-standards, accessed on 21 April 2021) SeraCare (https://www.seracare.com/Controls---Reference-Materials-NGS, accessed on 21 April 2021)
	Patient specimens	All processes from wet to dry bench is monitored Clinically relevant variants are included Specimens confirmed by other orthogonal molecular methods are needed Patient specimens are exhaustible	

Abbreviations: QC, quality control; VAF, variant allele frequency; SNV, single nucleotide variant; Indel, insertion and deletions; CNV, copy number variant.

## Data Availability

Not applicable.
